# A New Application for Cenicriviroc, a Dual CCR2/CCR5 Antagonist, in the Treatment of Painful Diabetic Neuropathy in a Mouse Model

**DOI:** 10.3390/ijms25137410

**Published:** 2024-07-05

**Authors:** Aleksandra Bober, Anna Piotrowska, Katarzyna Pawlik, Katarzyna Ciapała, Magdalena Maciuszek, Wioletta Makuch, Joanna Mika

**Affiliations:** Department of Pain Pharmacology, Maj Institute of Pharmacology, Polish Academy of Sciences, 31-343 Krakow, Poland; bober@if-pan.krakow.pl (A.B.); pawlik@if-pan.krakow.pl (K.P.); kat.ciapala@gmail.com (K.C.); maciuszekmagda@gmail.com (M.M.); makuch@if-pan.krakow.pl (W.M.)

**Keywords:** neuropathic pain, diabetes, cenicriviroc, chemokines, male, female

## Abstract

The ligands of chemokine receptors 2 and 5 (CCR2 and CCR5, respectively) are associated with the pathomechanism of neuropathic pain development, but their role in painful diabetic neuropathy remains unclear. Therefore, the aim of our study was to examine the function of these factors in the hypersensitivity accompanying diabetes. Additionally, we analyzed the analgesic effect of cenicriviroc (CVC), a dual CCR2/CCR5 antagonist, and its influence on the effectiveness of morphine. An increasing number of experimental studies have shown that targeting more than one molecular target is advantageous compared with the coadministration of individual pharmacophores in terms of their analgesic effect. The advantage of using bifunctional compounds is that they gain simultaneous access to two receptors at the same dose, positively affecting their pharmacokinetics and pharmacodynamics and consequently leading to improved analgesia. Experiments were performed on male and female Swiss albino mice with a streptozotocin (STZ, 200 mg/kg, i.p.) model of diabetic neuropathy. We found that the blood glucose level increased, and the mechanical and thermal hypersensitivity developed on the 7th day after STZ administration. In male mice, we observed increased mRNA levels of *Ccl2*, *Ccl5*, and *Ccl7*, while in female mice, we observed additional increases in *Ccl8* and *Ccl12* levels. We have demonstrated for the first time that a single administration of cenicriviroc relieves pain to a similar extent in male and female mice. Moreover, repeated coadministration of cenicriviroc with morphine delays the development of opioid tolerance, while the best and longest-lasting analgesic effect is achieved by repeated administration of cenicriviroc alone, which reduces pain hypersensitivity in STZ-exposed mice, and unlike morphine, no tolerance to the analgesic effects of CVC is observed until Day 15 of treatment. Based on these results, we suggest that targeting CCR2 and CCR5 with CVC is a potent therapeutic option for novel pain treatments in diabetic neuropathy patients.

## 1. Introduction

Diabetic neuropathy is a chronic disease resulting from nerve damage, and research has shown that it affects up to 50% of diabetic patients [1,2,3]. There is twice as much risk of developing pain in type 2 diabetic patients in comparison to type 1 diabetic patients [4]. The same risk evaluation applies to female versus male patients [4]. Painful symptoms are present in 26% of patients without neuropathy and in 60% of patients with severe neuropathy [4]. The FDA-approved drugs for treating diabetic neuropathy include anticonvulsants, antidepressants, and opioids [5], some of which can reduce pain levels, but the efficacy of these drugs is still unsatisfactory. Additionally, these drugs may be ineffective in advanced refractory patients or may cause adverse effects [6,7]. In the case of opioids, lower pain analgesia is observed due to tolerance development [7,8,9]. Recent studies have shown that immune factors, including chemokines, strongly increase neuropathic pain [10,11] and are responsible for reducing the effectiveness of morphine [12,13].

Chemokines are small (8–12 kDa) molecules with inflammatory and homeostatic properties, depending on the surrounding microenvironment [14]. A notable number of chemokines have been proven to have pronociceptive properties, and their involvement in pain transmission is well established [15,16,17,18,19,20,21,22]. This characteristic makes them great potential targets for novel therapies targeting neuropathic pain, as current analgesic treatments are insufficient and can cause adverse effects [23,24,25]. Research has shown that the administration of chemokine-neutralizing antibodies and chemokine receptor antagonists reduces the mechanical and thermal hypersensitivity evoked by nerve injury [15,16,17,26] and streptozotocin (STZ) [18,19,27]. Additionally, such treatments improve opioid efficacy [16,17,18,27,28]; this seems to be especially important because opioid receptors undergo chemokine-induced desensitization, which results in reduced analgesia [29]. An in vitro study showed that the chemotactic activities of two opioid receptors, namely, MOR and DOR, are decreased after activation of CCR2 and CCR5, which is most likely due to heterodimer formation between these receptors [29]. CCR2 and CCR5 share several ligands, namely, CCL7, CCL8, CCL11, and CCL16 (which are present only as pseudogenes in rodents [30,31]). The remaining CCR5 ligands are CCL3, CCL4, and CCL5, while the remaining CCR2 ligands are CCL2 and CCL13 (not present in mice) [31]. Both receptors are strongly involved in nociceptive transmission since their ligands are known to be responsible for the development (CCL2/3/5/7/8) and persistence (CCL2/7/8) of neuropathic pain [26]. Importantly, some chemokines, such as CCL2 [16], CCL3 [27], and CCL7 [16], decrease the analgesic effect of morphine, and the use of chemokine-neutralizing antibodies restores opioid analgesia. Furthermore, the strong analgesic effect of cenicriviroc (CVC), a dual CCR2/CCR5 antagonist, has already been proven in rat and mouse models of chronic constriction injury to sciatic nerve (CCI)-induced neuropathic pain [15,17]. Repeated intrathecal administration of CVC decreased the mRNA levels of several pronociceptive ligands of CCR2 and CCR5 in the spinal cord and dorsal root ganglia (DRG) in CCI-exposed rats [17].

Compared with RS504393 (a CCR2 antagonist) and maraviroc (a CCR5 antagonist), single [15,17] or repeated [17] intrathecal administration of CVC significantly decreased CCI-induced hypersensitivity in rats. Additionally, CVC had the most effective antinociceptive effect on mice after single intrathecal and intraperitoneal injections following CCI [17]. Moreover, the use of CVC enhances opioid analgesic potency [15,17], but its effect on diabetic neuropathic pain (DNP) is still unknown. Our previous study in an STZ model showed that blocking CCR4 [18] and CCR8 [19] decreases hypersensitivity and increases the effectiveness of morphine. Therefore, we investigated the effect of a dual CCR2/CCR5 antagonist on pain-related behaviors and the development of morphine tolerance in the STZ model. Moreover, since the studies thus far have primarily been conducted on males and painful diabetic neuropathy also often affects women, we decided to perform most of the experiments in both male and female mice.

Therefore, based on the literature mentioned above, our first aim was to examine the pain response in parallel with the spinal levels of two chemokine receptors (CCR2, CCR5) and their ligands (CCL2, CCL3, CCL4, CCL5, CCL7, CCL8, CCL11, CCL12) as well as opioid receptors (MOR, DOR, KOR) 7 days after STZ administration in male and female mice. Our second goal was to determine whether a single intraperitoneal injection of CVC in both sexes influences tactile and thermal hypersensitivity resulting from diabetes. Our third aim was to determine whether and how the repeated intraperitoneal injection of CVC influences tactile and thermal hypersensitivity as well as the analgesic properties of morphine in females with diabetic neuropathic pain. Such research is necessary because the role of chemokines and their receptors in diabetic neuropathic pain remains to be determined. Furthermore, discovering new therapeutic targets may contribute to the introduction of new drugs or the development of new ways to utilize existing drugs, such as cenicriviroc [32,33], for treating diabetic neuropathic pain. The present research is extremely important in the context of an increasing number of patients suffering from diabetes and the utilization of effective pharmacology to treat pain.

## 2. Results

### 2.1. The Effect of a Single STZ Administration on Weight, Blood Glucose Level, and Pain-Related Behavior on Day 7 after STZ Injection in Male and Female Mice

**Figure 1:**
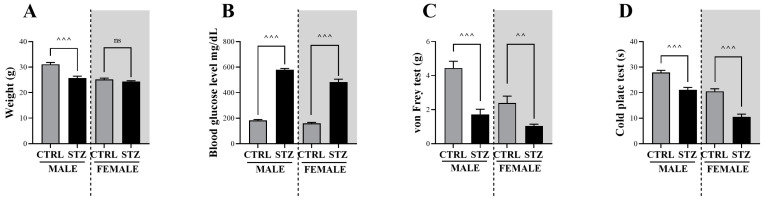
Changes in weight, blood glucose levels, and pain hypersensitivity on the 7th day after vehicle or STZ treatment in male and female mice. The effects of a single STZ injection on weight (**A**), blood glucose level (**B**), and pain hypersensitivity (**C**,**D**). The behavioral tests were performed on Day 7 after STZ injection. Mechanical and thermal hypersensitivity were measured by von Frey (**C**) and cold plate (**D**) tests, respectively. The data are presented as the mean ± SEM. The total number of animals included 70 mice: 32 males (*n* = 14–17 per group) and 38 females (*n* = 18–20 per group). The results were statistically evaluated using a *t*-test; ^^ *p* < 0.01 and ^^^ *p* < 0.001 indicate changes compared with non-diabetic mice injected with water. Abbreviations: ns, not significant; STZ, streptozotocin; CTRL, control.

On Day 7 after STZ administration, the weight of male diabetic mice was lower than that of non-diabetic mice (CTRL; ^^^ *p* < 0.001), but this trend was not observed in female diabetic mice (Figure 1A). On Day 7, blood glucose levels were greater in diabetic mice than in non-diabetic male (^^^ *p* < 0.001) and female (^^^ *p* < 0.001) mice (Figure 1B). Both male and female diabetic mice displayed mechanical (male: ^^^ *p* < 0.001, female: ^^ *p* < 0.01; Figure 1C) and thermal (male/female: ^^^ *p* < 0.001; Figure 1D) hypersensitivity.

### 2.2. The Effect of a Single STZ Administration on the mRNA Levels of CCR2 and CCR5 Ligands Measured on Day 7 after STZ Injection in Male and Female Mice

**Figure 2:**
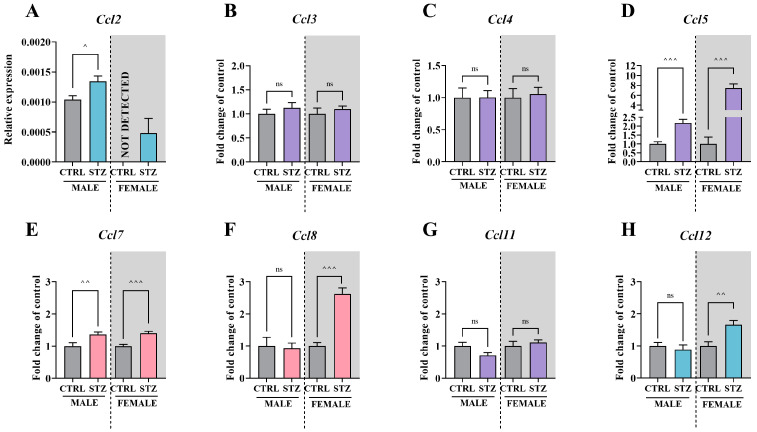
Changes in the mRNA levels of CCR2 and CCR5 ligands on the 7th day after vehicle or STZ treatment in male and female mice. The effects of a single STZ injection on the mRNA levels of *Ccl2* (**A**), *Ccl3* (**B**), *Ccl4* (**C**), *Ccl5* (**D**), *Ccl7* (**E**), *Ccl8* (**F**), *Ccl11* (**G**), and *Ccl12* (**H**) were measured by RT–qPCR. The data are presented as the fold change relative to that of control mice (non-diabetic mice injected with water for injection) ± SEM, except for (**A**), where data are presented as a relative expression ± SEM. The total number of animals was 43 mice, with 23 males (*n* = 10–12 per group) and 20 females (*n* = 8–11 per group), except for (**D**), in which females (*n* = 5–8 per group) were included. The results were statistically evaluated using a *t*-test; ^ *p* < 0.05, ^^ *p* < 0.01, and ^^^ *p* < 0.001 indicate changes compared with the control. Abbreviations: ns, not significant; STZ, streptozotocin; CTRL, control.

On Day 7 after STZ administration, the spinal mRNA levels of *Ccl2* (^ *p* < 0.05; Figure 2A), *Ccl5* (^^^ *p* < 0.001; Figure 2D), and *Ccl7* (^^ *p* < 0.01; Figure 2E) were increased in male diabetic mice, while in female diabetic mice, an increase in *Ccl5* (^^^ *p* < 0.001; Figure 2D), *Ccl7* (^^^ *p* < 0.05; Figure 2E), *Ccl8* (^^^ *p* < 0.001; Figure 2F) and *Ccl12* (^^ *p* < 0.01; Figure 2H) were detected. Notably, in female mice, *Ccl2* mRNA was not detected in three out of 11 in the diabetic group and seven out of nine individuals in the non-diabetic group; therefore, on the graph, it is marked as undetected (Figure 2A). There were no statistically significant differences in *Ccl3* (Figure 2B), *Ccl4* (Figure 2C), and *Ccl11* (Figure 2G) between diabetic and control groups in both male and female mice.

### 2.3. The Effect of a Single STZ Administration on the Protein Levels of CCR2 and CCR5 Ligands (CCL2, CCL5, CCL7, and CCL8) Measured on Day 7 after STZ Injection in Male and Female Mice

**Figure 3:**
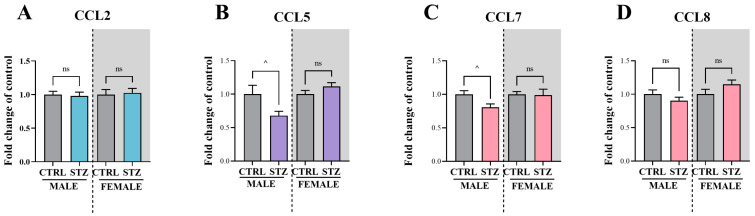
Changes in the protein levels of CCR2 and CCR5 ligands (CCL2, CCL5, CCL7, and CCL8) on the 7th day after vehicle or STZ treatment in male and female mice. The effects of a single STZ injection on the protein levels of CCL2 (**A**), CCL5 (**B**), CCL7 (**C**), and CCL8 (**D**) were measured by ELISA. The data are presented as the fold change relative to that of control mice (non-diabetic mice were injected with water for injection) ± SEM. The total number of animals was 40 mice: 20 males (*n* = 7–10 per group) and 20 females (*n* = 8–10 per group). The results were statistically evaluated using a *t*-test; ^ *p* < 0.05 indicates changes compared with the control. Abbreviations: ns, not significant; STZ, streptozotocin; CTRL, control.

On Day 7 after STZ administration, the spinal cord protein levels of CCL5 (^ *p* < 0.05; Figure 3B) and CCL7 (^ *p* < 0.05; Figure 3C) were decreased in male diabetic mice, and no changes were observed in the CCL2 and CCL8 levels; moreover, in female diabetic mice, no changes in CCL2 (Figure 3A), CCL5 (Figure 3B), CCL7 (Figure 3C) or CCL8 (Figure 3D) were detected.

### 2.4. The Effect of a Single STZ Administration on the CCR2 and CCR5 mRNA and Protein Levels Measured on Day 7 after STZ Injection in Male and Female Mice

**Figure 4:**
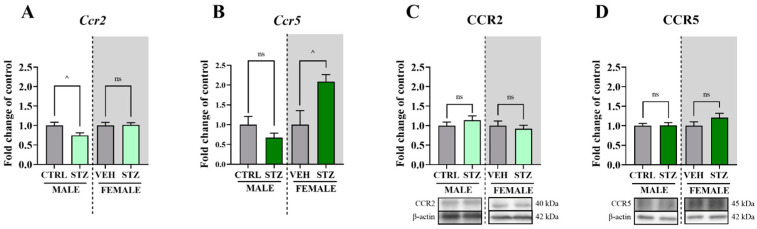
Changes in the CCR2 and CCR5 mRNA and protein levels on the 7th day after vehicle or STZ treatment in male and female mice. The effects of a single STZ injection on *Ccr2* and *Ccr5* mRNA (**A**,**B**) and CCR2 and CCR5 protein (**C**,**D**) levels were measured by RT-qPCR and Western blot, respectively. The data are presented as the fold change relative to that of control mice (non-diabetic mice were injected with water for injection) ± SEM. The total number of animals was 43 mice: 23 males (*n* = 9–12 per group) and 20 females (*n* = 7–11 per group). The results were statistically evaluated using a *t*-test; ^ *p* < 0.05 indicates changes compared with the control. Abbreviations: ns, not significant; STZ, streptozotocin; CTRL, control.

On Day 7 after STZ administration, the spinal cord *Ccr2* mRNA levels were decreased in male diabetic mice (^ *p* < 0.05), but no changes were observed in female diabetic mice (Figure 4A). *Ccr5* mRNA levels were increased in female diabetic mice (^ *p* < 0.05), but no significant changes were observed in male diabetic mice (Figure 4B). STZ administration did not influence protein levels of CCR2 and CCR5 in both sexes (Figure 4C,D).

### 2.5. The Effect of a Single STZ Administration on the mRNA Levels of Opioid Receptors Measured on Day 7 after STZ Injection in Male and Female Mice

On Day 7 after STZ administration, the spinal mRNA levels of *Oprm1*, *Oprd1,* and *Oprk1* were unaffected in female diabetic mice compared with those in control mice. *Oprm1* and *Oprd1* mRNA levels were decreased (^ *p* < 0.05 and ^^ *p* < 0.01, respectively) in STZ-induced male mice, but no change in *Oprk1* mRNA level was detected compared with those in control mice (Table 1).

### 2.6. The Effect of a Single Intraperitoneal Injection of Cenicriviroc on Hypersensitivity Measured 7–8 Days after STZ Injection in Male and Female Mice

**Figure 5:**
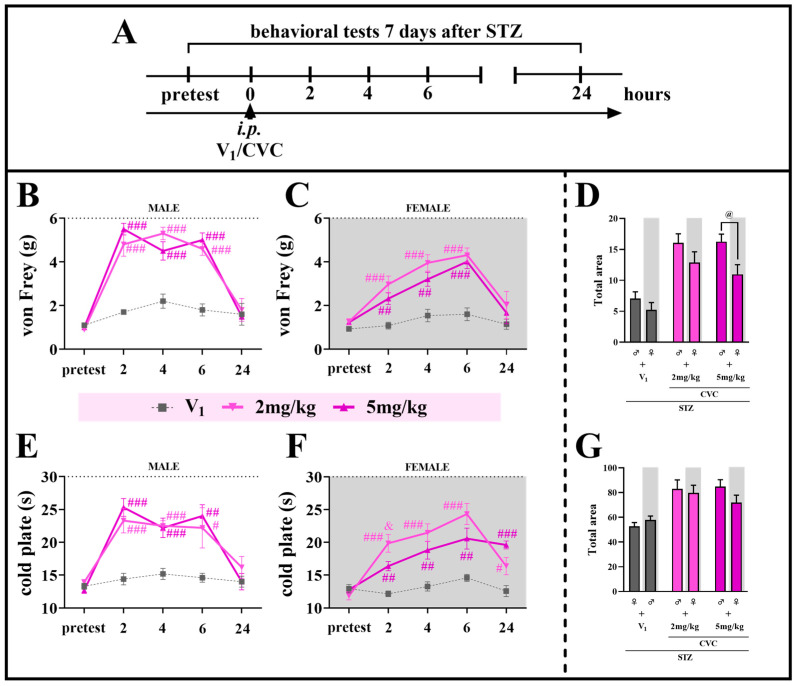
Effect of i.p. injection of CVC on pain hypersensitivity measured on the 7th day after vehicle or STZ treatment in male and female mice. Pretests were performed before CVC administration, and behavioral tests were performed 2, 4, 6, and 24 h after CVC injection (**A**). Thermal and mechanical hypersensitivity were measured by von Frey (**B**,**C**) and cold plate (**E**,**F**) tests, respectively. The AUC was calculated for each test (**D**,**G**). The data are presented as the mean ± SEM. The total number of animals: 64 mice: 23 males (*n* = 7–8 per group at each time point) and 41 females (*n* = 11–15 per group at each time point except 24 h, where *n* = 4–6 per group). The results were statistically evaluated using one-way ANOVA with the Bonferroni correction; ^#^ *p* < 0.05, ^##^ *p* < 0.01, and ^###^ *p* < 0.001 indicate changes compared with those in the V_1_ group, ^&^ *p* < 0.05 indicates changes between mice treated with 2 and 5 mg/kg CVC, and ^@^ *p* < 0.05 indicates changes between male and female mice. Abbreviations: AUC, area under the curve; CVC, cenicriviroc; intraperitoneal, i.p.; STZ, streptozotocin; V_1_, vehicle (1% DMSO).

On Day 7 after STZ administration in diabetic mice (Figure 5A), CVC at doses of 2 mg/kg and 5 mg/kg alleviated the mechanical (^###^ *p* < 0.001 in each study group; Figure 5B) and thermal (Figure 5E) hypersensitivity in male mice 2, 4, and 6 h after the CVC injection compared with that in the V_1_-treated group. Additionally, in diabetic female mice, both doses significantly alleviated the mechanical (Figure 5C) and thermal (Figure 5F) hypersensitivity at 2, 4, 6, and even 24 h (for thermal hypersensitivity) after the CVC injection compared with that in the V_1_-treated group. The area under the curve (AUC) analysis of the obtained von Frey test data showed that the highest dose (5 mg/kg) at all tested time points was more effective in males than in females (Figure 5D). No statistically significant differences between male and female were noted in the AUC of the cold plate test (Figure 5G). Two-way ANOVA confirmed a significant interaction between both the CVC doses and the tested time points in the von Frey and cold plate tests (*p* = 0.0007 and *p* < 0.0001, respectively).

### 2.7. The Effect of Repeated Two Daily Intraperitoneal Injections of Cenicriviroc (CVC) on Hypersensitivity Development and the Analgesic Effect of Morphine Were Measured between 8 and 22 Days after STZ Injection in Female Mice

**Figure 6:**
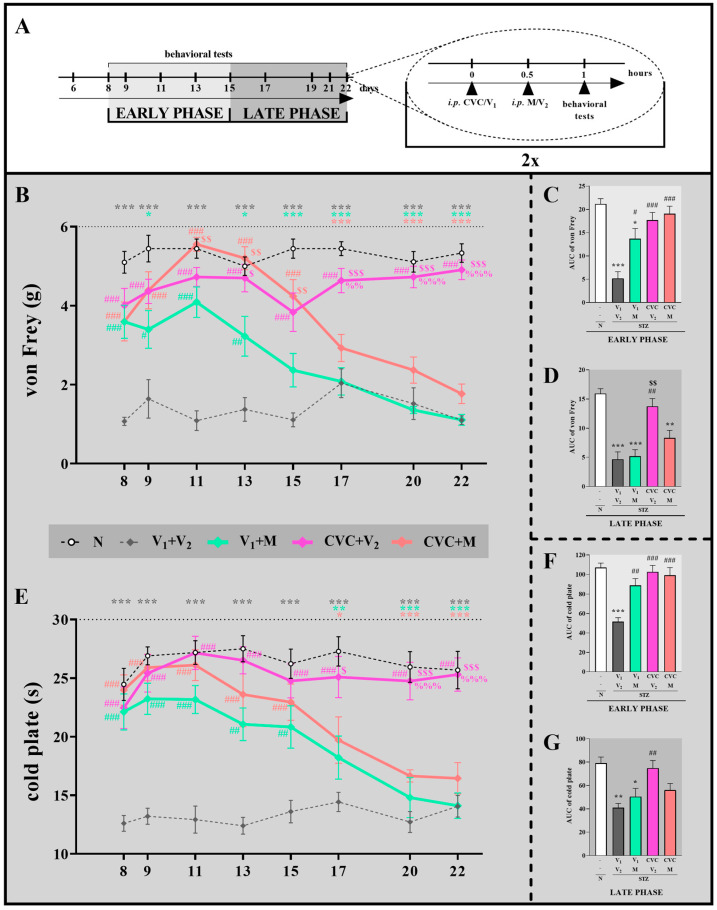
Effect of repeated i.p. CVC injections on pain hypersensitivity and morphine tolerance on Days 6–22 after STZ treatment in female mice. Pretests were performed on Day 6 after STZ injection. (**A**). Behavioral tests were performed on Days 8, 9, 11, 13, 15, 17, 20, and 22. In diabetic mice V_2_ or M was administered twice daily 0.5 h after V_1_ or CVC injection, and behavioral tests were performed 0.5 h after the last injection (**A**). Thermal and mechanical hypersensitivity were measured by von Frey (**B**) and cold plate (**E**) tests, respectively. The early (Days 8–15) and late (Days 15–22) phase AUC were calculated (**C**,**D**,**F**,**G**). The data are presented as the mean ± SEM. The total number of animals: 57 mice, *n* = 9–12 per group. The results were statistically evaluated using one-way ANOVA with the Bonferroni correction; * *p* < 0.05, ** *p* < 0.01, and *** *p* < 0.001 indicate changes compared with the naïve group; ^#^ *p* < 0.05, ^##^ *p* < 0.01, and ^###^ *p* < 0.001 indicate changes compared with the V_1_ + V_2_ group; ^$^ *p* < 0.05, ^$$^ *p* < 0.01, and ^$$$^ *p* < 0.001 indicate changes compared with the V_1_ + M-group; and ^%%^ *p* < 0.01 and ^%%%^ *p* < 0.001 indicate changes compared with the CVC + M group. Abbreviations: AUC, area under the curve; CVC, cenicriviroc; intraperitoneal, i.p.; STZ, streptozotocin; V_1_, vehicle (1% DMSO); V_2_, vehicle (water for injection); M, morphine; N, naïve.

Administration of the tested substances started on Day 6 after STZ administration (Figure 6A). The administration of STZ significantly decreased the reaction threshold to tactile and thermal stimuli in the tested female mice, indicating the development of hypersensitivity. Compared with the naïve animals, the STZ-exposed group treated with V_1_ + V_2_ showed pain-like behavior, manifested by a significantly (*** *p* < 0.001) reduced response threshold to tactile (Figure 6B) and thermal (Figure 6E) stimuli at all time points tested (both in the early, Days 8–15 and later, Days 15–22, phases of administration). By Day 11, all test groups showed a significant difference compared with the V_1_ + V_2_ group, as shown by both the von Frey and cold plate tests, which indicates the analgesic effect of the used substances. From this time point, the analgesic effectiveness of morphine consistently decreased, and consequently, the reaction threshold in groups that received morphine (V_1_ + M; CVC + M) did not differ significantly from that in vehicle-treated mice (V_1_ + V_2_) in the von Frey test from Day 15 (for the V_1_ + M group) or 17 (for the CVC + M group) or in the cold plate test from Day 17 (in both groups), which indicates the development of tolerance to morphine.

Interestingly, in the group receiving only cenicriviroc (CVC + V_2_), no decrease in the effectiveness of its analgesic effect was observed at any time point tested up to the last 22nd day of the study. For the whole duration of the experiment, the CVC + V_2_-administered group showed reduced mechanical and thermal hypersensitivity compared with that of the V_1_ + V_2_ group, and in the late phase of the study, it also significantly differed from the remaining study groups.

Additionally, the AUC analysis of the data obtained from the von Frey and cold plate tests showed that in the first phase, all administered substances reduced mechanical (Figure 6C) and thermal (Figure 6F) hypersensitivity in the V_1_ + V_2_ group. However, in the late phase, the AUC analysis indicated that only CVC administration was effective in diabetic females (Figure 6D,G). Two-way ANOVA confirmed a significant interaction between the investigated treatment and the investigated time points in the von Frey and cold plate tests (*p* < 0.0001).

### 2.8. Effect of Repeated Two Daily Intraperitoneal Injections of Cenicriviroc (CVC) on Weight, Blood Glucose Levels, and Motor Performance in Female Mice 21–22 Days after STZ Injection

**Figure 7:**
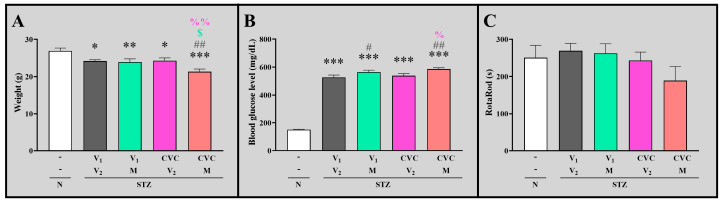
Effect of repeated i.p. CVC injections on weight, blood glucose levels, and motor functions on Days 21–22 after STZ treatment in female mice. Weight (**A**) and blood glucose level (**B**) were measured on the last (22) day of behavioral testing. Motor functions were measured on Day 21, 0.5 h after morphine injection by the rotarod test (**C**). The data are presented as the mean ± SEM. The results were statistically evaluated using one-way ANOVA with the Bonferroni correction; * *p* < 0.05, ** *p* < 0.01, and *** *p* < 0.001 indicate changes compared with the naïve group; ^#^ *p* < 0.05 and ^##^ *p* < 0.001 indicate changes compared with the V_1_ + V_2_ group; ^$^ < 0.05 indicates changes compared with the V_1_ + M-group; and ^%^ *p* < 0.05 and ^%%^ *p* < 0.01 indicate changes compared with the CVC + M group. Abbreviations: CVC, cenicriviroc; intraperitoneal, i.p.; STZ, streptozotocin; V_1_, vehicle (1% DMSO); V_2_, vehicle (water for injection); M, morphine; N, naïve.

On Day 22 after STZ injection, a significant decrease in weight (Figure 7A) and increased blood glucose were observed (Figure 7B) in all studied groups of diabetic mice compared with the naïve mice. Moreover, on Day 21, no changes in motor functions were observed in the individual diabetic groups compared with the naïve group (Figure 7C). Compared with all the other groups, the CVC + M-treated group exhibited significantly decreased body weight and the highest blood glucose level. This was the only group in which we observed impaired motor functions, although this finding was not statistically significant.

## 3. Discussion

Our research revealed that at the spinal cord level, the expression of the endogenous CCR2 (CCL2/7/8) and CCR5 (CCL5/7/8) ligands, which have known pronociceptive properties, was significantly upregulated at the mRNA level 7 days after STZ administration. Importantly, our pharmacological studies have proven that CCR2 and CCR5 play significant roles in the development of pain hypersensitivity resulting from diabetes. For the first time using the painful diabetic neuropathy model, we report the beneficial effects of cenicriviroc, a dual CCR2/5 antagonist. The analgesic effects of cenicriviroc were observed after single and/or repeated injections in the early and late phases of painful diabetic neuropathy in a mouse model. Additionally, this dual antagonist diminished the mechanical and thermal hypersensitivity evoked by STZ administration in both male and female mice. Moreover, the presented pharmacological results demonstrated that coadministration of cenicriviroc and morphine slightly delayed the development of tolerance to the antinociceptive effect of morphine. However, when administered alone, cenicriviroc is more effective at providing analgesia than morphine alone or in combination with morphine.

STZ-evoked diabetes is a well-known animal model used in many neuropathic pain studies [34,35,36,37,38], including our previous research [18,19,20,27,39]. Hyperglycemia is responsible for disruptions in Schwann cell metabolic activity and myelin degeneration, resulting in the development of neuropathic pain [40,41,42]. The reaction to noxious stimuli is conducted by lightly myelinated Aδ and unmyelinated C fibers, whereas the response to non-noxious stimuli is conducted by highly myelinated Aβ fibers [43]. Currently, the neuroimmunological background of diabetic neuropathic pain is complex [18,19,20,27,39,40,43,44]; however, it is still not fully understood. In the STZ model, increased blood glucose concentrations correlate with weight loss and hypersensitivity to tactile and thermal stimuli [18,20,39]. For biochemical and pharmacological studies, we selected Day 7 after STZ injection since, at that time point, pain-related behavior is fully developed [39]. For several years, increasing evidence has emphasized the role of chemokines in the pathology of neuropathic pain [19,20,27,39,44]. However, the role of CCR2 and CCR5 in diabetic neuropathy remains to be determined, which is why we focused on those receptors in our research.

CCR2 is expressed in spinal microglia [45], astrocytes [46], and neurons [47], and its involvement in the development of neuropathic pain of different etiologies is well established in the literature [21,48,49]. For example, CCR2 knockout mice exhibit delayed hypersensitivity up to 15 days after nerve injury [45]. It was also shown that compression of the DRG (L4/L5) caused *Ccr2* mRNA upregulation in neuronal and non-neuronal cells [50]. Moreover, the upregulation of spinal or DRG CCR2 mRNA and/or protein level was detected 2–14 days after CCI in rats [51]. However, in a diabetic neuropathic pain model, *Ccr2* mRNA was upregulated in the sciatic nerve but not in the spinal cord in rats [52,53]. These results are consistent with our findings, which show that *Ccr2* mRNA levels do not change on Day 7 at the spinal cord level in STZ-induced diabetic female mice and even decrease in male mice. Meanwhile, we did not observe a change in CCR2 protein levels in male and female mice. Interestingly, in type 2 diabetic monkeys, *Ccr2* mRNA levels are increased in the spinal cord [54]. Notably, clinical studies have shown that CCR2 expression is increased in monocytes isolated from the blood of type 2 diabetes patients [55]. In summary, the literature indicates that CCR2 may play an important role in the periphery of hypersensitivity resulting from diabetes, but this topic requires further detailed studies. In mice, several chemokines, including CCL2, CCL7, CCL8, and CCL12, bind to CCR2, all except CCL12, having strong pronociceptive properties [56,57,58]. The main ligand of this receptor is CCL2, which, after intrathecal administration, induces long-lasting tactile and thermal hypersensitivity in mice [16,26,47], and its role in neuropathic pain is well established [51,59,60]. CCL2 is a strong chemoattractant for leukocytes [61,62,63,64]. Moreover, CCL2 can be released as part of the extracellular vesicles package and, because of that, also has a role in neuroinflammation and neurodegeneration [65,66]. Recently, it was proven that the CCL2-CCR2 axis is important in diabetes, especially for monocyte recruitment [67]. Moreover, long-term changes in CCL2 levels were also detected in male rat [68] and mouse [69] models of diabetes. Additionally, type 2 diabetic monkeys showed increased spinal *Ccl2* mRNA levels [54]. Similarly, the upregulation of spinal *Ccl2* mRNA in male diabetic mice was observed in our study, which agrees with previously published results [18,68,69]. In naïve female mice, *Ccl2* mRNA was undetectable; however, after STZ administration, a certain level of *Ccl2* mRNA was detected. Importantly, in clinical studies, elevated CCL2 levels were detected in the blood of patients with type 1 [70] and type 2 [71] diabetes. Based on our study and available data, we hypothesize that the CCL2-CCR2 axis may be a potential pharmacological target in males and likely also in females. Further research is needed to fully understand and establish the role of CCL2 in the progression of DNP in both sexes, especially in clinical trials.

CCR5 is also known to be an important receptor for pain signaling transmission [28,72,73], as it is a target of pronociceptive chemokines, including CCL3, CCL4, CCL5, CCL7, CCL8, and CCL11 [26,27,31,58]. It has already been shown that the spinal mRNA/protein levels of CCR5 are increased in CCI-exposed rats [28] and type 2 diabetic monkeys [54]. However, similar to CCR2, *Ccr5* mRNA was increased in the sciatic nerve but not in the spinal cord of STZ-induced diabetic rats [52]. In male diabetic mice, the spinal CCR5 mRNA/protein level did not change 7 days after STZ injection, as shown in our previous study [27]. For the first time, we showed that *Ccr5* mRNA levels are increased in female mice, however, protein level remains unchanged. Recently, flow cytometry analysis revealed that the CCR5 lymphoid ratio is increased in diabetic patients with a disease course of less than 5 years, and both the lymphoid and mononuclear CCR5 ratios are increased in diabetic patients with a disease course of more than 5 years [74]. Our study demonstrated differences in CCR5 levels between male and female mice, which suggests that further research on humans needs to focus on confirming sex differences and implementing appropriate treatments for male and female patients. CCL5 is the main ligand of CCR5 [10] and is known to recruit immune cells to the inflammation site [75]. CCL5 is involved in nociception, as several studies have proven that its level is altered in neuropathic pain of different etiologies [76,77,78,79]; for instance, the *Ccl5* spinal mRNA level is upregulated 7–14 days after CCI in mice [26]. Moreover, a single intrathecal injection of CCL5 evoked mechanical and thermal hypersensitivity in naïve mice [26]. Recently, it was also shown that some CCL5 gene polymorphisms are associated with a greater risk of developing diabetes [80,81,82]; however, no data about the contribution of CCL5 to diabetic neuropathic pain exist. Our findings indicate for the first time that on Day 7 after STZ injection, the spinal *Ccl5* mRNA levels are upregulated in male and female mice. A study performed on patients with type 1 diabetes showed that CCL5 levels are elevated in blood samples [70], which was subsequently confirmed by a meta-analysis [83]. We suggest that, similarly to CCL2-CCR2 axis, CCL5-CCR5 interactions might be meaningful for diabetic neuropathic pain development in both male and female mice, however, further research is needed to confirm this hypothesis.

CCR2 and CCR5 share two pronociceptive ligands, CCL7 [16,26] and CCL8 [26]; therefore, blocking both receptors simultaneously seemed to be an interesting strategy for alleviating pain in diabetic neuropathy. CCL7 shares 60–71% [84] and CCL8 shares 62.5% [85] homology with CCL2. Similarly to CCL2, the intrathecal administration of CCL7 and CCL8 causes mechanical and thermal hypersensitivity in naïve mice [26]. CCL7 was shown to contribute to hypersensitivity development in pNSL-induced [86], SNL-induced [87], and CCI-induced [16,26] neuropathic pain, and CCL7-neutralizing antibodies were shown to reduce neuropathic pain symptoms in a CCI model [16]. Additionally, CCL7 knockout mice develop neuropathic pain to a lesser extent [87]. In our current study, we observed enhanced spinal *Ccl7* mRNA levels in male and female mice 7 days after STZ administration. Similarly, upregulated *Ccl7* mRNA levels were also observed in the pancreatic islets of transgenic NOD mice with cyclophosphamide-induced diabetes [88]. Importantly, it was also shown that the CCL7 level is increased in monocytes derived from the blood of type 1 and type 2 diabetic patients [71]. Based on the literature, CCL7 seems to be an important factor responsible for the development of neuropathic pain [86,87], and our results prove its contribution to hypersensitivity resulting from diabetes. CCL8, like CCL2 and CCL7, can attract immune cells, mainly monocytes [85]. Upregulated CCL8 mRNA/protein spinal levels are found shortly after CCI and can be detected even up to 28 days after surgery in a mouse neuropathic pain model [26]. Our findings showed for the first time that, in a diabetic neuropathic pain model, the spinal level of *Ccl8* mRNA did not change in male diabetic mice but significantly increased in female diabetic mice on Day 7 after STZ administration. In the clinic, increased *CCL8* mRNA levels were also observed in ex vivo TCR-induced leukocytes isolated from the blood of type 1 diabetic children [89], and elevated CCL8 protein levels were detected in the aqueous humor of patients with type 2 diabetes [90]; however, sex differences were not considered. Based on the literature and our data, we suggest that CCL8 might be an important factor in the development and progression of diabetic neuropathy and in the differences in experiencing painful symptoms between men and women, although further research is needed on this topic. Diabetes is a very complex disease. Its pathomechanism may also include metabolic memory [91,92] or the release of neutrophil extracellular traps (NETs) [93,94,95,96,97]. That is why we can also hypothesize that increased expression of *Ccl2*, *Ccl7*, or *Ccl8* may also cause increased formation of NETs and participate in various metabolic changes, which, in turn, contribute to severe diabetic complications. However, possible contribution of these mechanisms needs further research and explanation.

In most cases of neuropathic pain, women more often experience pain symptoms than men do [98,99]. Research on the diabetic population in the United Kingdom showed that women had a 50% increased risk of developing painful symptoms compared with men [4]. Although a more recent study conducted in Canada showed that diabetic neuropathic pain is more prevalent in males, women report painful symptoms more often [100], and this pain is usually greater than that in men [101]. The relationships among neurons, glia, and the immune system have attracted increasing attention as potential causes of differences in pain hypersensitivity between males and females [98]. The morphology and function of microglia and astrocytes differ between sexes [102], as does the expression of chemokines and chemokine receptors. Research has shown that chemokines such as CCL2 [103], CCL3, and CCL4 [104] are differentially expressed in female versus male mouse brain tissue. Analysis of mesenteric tissues and leukocytes from the peritoneal cavity showed that in female mice, the expression of CCL2 and CCL5 was increased compared with that in male mice. Additionally, the expression of some chemokines and their receptors changes after ovariectomy [105]. Moreover, research has shown that the percentage of astrocytes expressing CCL2, which is involved in the recruitment of immune cells and gliosis regulation, is greater in males than in females [103]. Recently, it was also demonstrated that in sciatic nerves, 7 days after CCI, CCL2 upregulation is observed in male mice, while CCL5 levels are increased in female mice [106]. To our knowledge, no data are currently available regarding sex differences in CC chemokine expression in DNP models or patients. Our study, for the first time, shows that at the spinal cord level, 7 days after STZ administration, when tactile and thermal hypersensitivity develop, the expression levels of *Ccl5* and *Ccl7* are increased in both male and female mice. Interestingly, in diabetic males, the expression of *Ccl2* was upregulated, whereas in females, *Ccl2* was detectable only after STZ treatment. In addition, in female mice, an increase in *Ccl8* and *Ccl12* levels is observed. CCL12 is the only known CCR2 ligand that does not show pronociceptive effects after intrathecal administration [16]. However, all other chemokines that changed at the mRNA level in our study may play important roles in the CNS [107] and probably also in the PNS [108]. More studies on the role of CCR2 and CCR5 ligands are needed since we did not observe statistically significant changes in CCL2 and CCL8 protein levels in either sex; nevertheless, blocking these chemokine receptors had significant analgesic effects. Thus far, it has been postulated that chemokines, for example, CCL2, are mainly produced in DRG neurons and then transported to the spinal cord [21,108]. All of the discussed results suggest that further long-term research investigating the spinal, DRG, and blood levels of chemokines is needed to thoroughly examine the role of chemokines in the development and maintenance of painful diabetic neuropathy. Although there are ambiguous and sometimes contradictory results regarding the expression levels of CCR2 and CCR5 and their ligands in diabetes, the literature and our findings still point out the significant role of these receptors in the development of neuropathic pain and suggest that these two receptors are suitable targets for novel pain therapies for the treatment of painful diabetic neuropathy in males and females.

Therefore, in the second part of our research, we conducted a pharmacological study using the STZ model in both sexes. The selective CCR2 (RS504393) and CCR5 (maraviroc) antagonists administered intrathecally attenuate pain-related symptoms in CCI rat and mouse models [28,51,109]. Nevertheless, the analgesic potential of a dual CCR2/5 antagonist (cenicriviroc) administered intrathecally or intraperitoneally is greater than that of single CCR2 or CCR5 antagonists, as measured in a mouse CCI-induced neuropathy model [17]. Furthermore, compared with those of RS504393 and maraviroc, the strongest molecular changes were observed after repeated intrathecal administration of cenicriviroc. Cenicriviroc downregulated the mRNA and/or protein levels of pronociceptive cytokines such as IL-1beta, IL-6, IL-18, CCL2, CCL3, CCL4, CCL5, and CCL7 in the spinal cord and/or DRG in CCI-exposed rats [15,17]. In the present study, we demonstrated for the first time that cenicriviroc also reduces mechanical and thermal hypersensitivity in male and female mice with STZ-induced neuropathic pain. These findings provide the first evidence that cenicriviroc is a potent analgesic agent for DNP in both sexes. Importantly, cenicriviroc is already undergoing clinical investigation in phase 2b and three trials for the treatment of patients with HIV infection [33] and liver fibrosis with nonalcoholic steatohepatitis [32,110], respectively. Therefore, our results are valuable in terms of reducing costs related to the design and testing of new drugs because cenicriviroc is an existing drug that might be introduced to a broader spectrum of therapies, including painful diabetic neuropathy treatment. Additionally, several chemokine receptor antagonists are already used in the clinic, e.g., AMD3100 (Plerixafor), a CXCR4 antagonist, is a clinically approved drug for the mobilization of hematopoietic stem cells for transplantation in patients with Non-Hodgkin’s lymphoma [111,112]. That is why we believe that blockades of chemokine receptors, including double blockades of CCR2 and CCR5, may be effectively used in the future to treat patients.

The treatment of neuropathic pain usually involves the coadministration of several drugs due to the complex pathomechanism and the relatively poor effectiveness of opioids [11,113]. One of the mechanisms responsible for the weakening effect of opioids in neuropathy is the downregulation of opioid receptors [114,115]. In the current study, we showed a decrease in the mRNA expression of *Mor* and *Dor* in the spinal cord of male mice exposed to STZ and a similar trend in female mice, which indicates the need to identify a strategy to counteract this phenomenon in a diabetic neuropathy model. Therefore, the aim of our further experiments was to determine the legitimacy of the use of multiple coadministration of cenicriviroc with morphine. According to the literature, repeated intrathecal administration of cenicriviroc enhances the analgesic effects of a single injection of morphine in rats with CCI-induced neuropathic pain [15]. Moreover, it was shown that single intraperitoneal administration of cenicriviroc enhanced morphine analgesia in a mouse CCI model [17], but the analgesic effect of repeated coadministration of these two drugs has not yet been investigated in diabetic neuropathy. However, it has already been revealed that the analgesic properties of morphine depend on the degree of spinal activation of microglia, i.e., the cells responsible for the release of numerous pronociceptive factors in neuropathy, including chemokines, which are endogenous ligands of CCR2 and CCR5 [10,116,117]. For instance, CCL2 neutralization diminishes morphine-induced microglial activation and enhances morphine analgesia [16,118]. In addition, CCR2 and CCR5, similar to opioid receptors (MOR and KOR), are present in neurons and glial/immune cells [10,113,119,120], and morphine can increase the levels of these chemokine receptors [121]. Moreover, CCR5 can form heterodimers with CCR2 [122] and MOR [123]. Therefore, the idea of using a bifunctional compound (cenicriviroc) as a drug to enhance the analgesic effect of morphine was developed. It is well known that in neuropathy, repeated administration of morphine is associated with many possible side effects, including the development of opioid tolerance [124,125]. Since pain affects women to a greater extent, and CVC provided pain relief to a similar extent in both sexes, we chose female mice for the repeated drug administration study, and we observed a gradual decrease in morphine analgesic potency after long-term daily administration. Our results provide the first evidence that repeated intraperitoneal injection of cenicriviroc delays the development of morphine tolerance, but the analgesic effect of coadministration of these drugs decreases over time. Although the mechanisms underlying this phenomenon are not fully understood, we assume that cenicriviroc influences the levels of pronociceptive cytokines, which are known to be responsible for the loss of opioid analgesia in neuropathy [10,11,113]. We also hypothesize that the observed result might be associated with heterologous desensitization between opioid and chemokine receptors [123,126,127]. Additionally, we also observed that the combined administration of cenicriviroc and morphine contributed to the greatest weight loss and increase in blood glucose compared with those of the other study groups, which is an interesting direction for further research to elucidate the mechanism of their combined action. Furthermore, our research proved that repeated administration of cenicriviroc alone is more effective at providing analgesia than treatment with morphine alone or morphine coadministered with cenicriviroc, which is especially important because the use of opioids in treatment is often insufficient and carries a risk of addiction and substance abuse [6,7]. Additionally, we wanted to determine whether the tested treatments influenced the motor functions of the mice, but we did not observe any differences between the study groups, which proves that cenicriviroc is safe for use in terms of motor control. This result is significant, as long-term morphine use has a negative impact on cognitive and motor functions in humans [128,129].

## 4. Materials and Methods

**Scheme 1:**
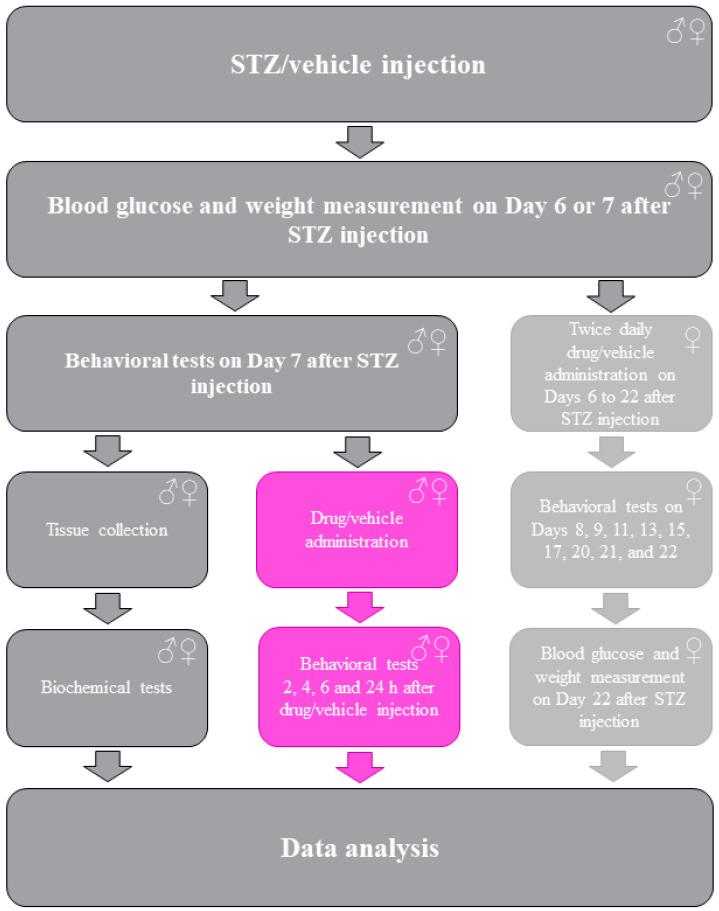
Study design and procedures used in the experiment.

Experiments were performed accordingly to experimental pipeline showed in Scheme 1.

### 4.1. Animals

Experiments were conducted on male (18–20 g) and female (13–20 g) Swiss albino mice purchased from Charles River (Sulzfeld, Germany). The mice were housed in cages with sawdust bedding on a standard 12 h/12 h light/dark cycle (lights on at 06:00 a.m.). Food and water were available ad libitum. All experiments were performed according to the International Association for the Study of Pain rules [130] and the National Institutes of Health Guide for the Care and Use of Laboratory Animals and were approved by the Ethical Committee of the Maj Institute of Pharmacology of the Polish Academy of Sciences (Krakow, Poland; permission numbers 43/2020, 111/2023 and 103/2024). The number of animals was reduced to the essential minimum according to the 3R policy—the total number of mice used in the study was 399 (120 males and 279 females).

### 4.2. Streptozotocin-Induced Diabetic Neuropathic Pain Model

Streptozotocin (STZ; 200 mg/kg; Sigma-Aldrich, St. Louis, MO, USA) was suspended in water for injection and administered by a single intraperitoneal (i.p.) injection [27]. Age-matched non-diabetic mice were injected with water (CTRL). Glucose concentrations were evaluated on Day 5 or 6 after STZ injection using an Accu-Chek active glucometer (Warsaw, Poland). Cut-off value of the measurement was 600 mg/dL. Mice were considered diabetic when their serum glucose levels exceeded 300 mg/dL. Body weight measurements were performed on Day 6, and specific behavioral tests were always performed by the same experimenter on Day 6 or 7 after STZ administration.

### 4.3. Behavioral Tests

#### 4.3.1. Mechanical Nociceptive Threshold—von Frey Test

The mechanical nociceptive threshold was evaluated with calibrated nylon monofilaments (Ugo Basile, Gemonio, Italy) according to methods described in our previous studies [27,131]. Von Frey filaments (with strengths ranging from 0.6 to 6 g) were applied consecutively to the plantar surface of both hind paws until withdrawal responses were observed.

#### 4.3.2. Thermal Nociceptive Threshold—Cold Plate Test

The thermal nociceptive threshold was evaluated using the cold plate test (Ugo Basile, Gemonio, Italy) as described in our previous studies [26,131]. A cold plate was used at a temperature of 2 °C, and the time until hind paw elevation was noted. The cutoff latency was set to 30 s.

#### 4.3.3. Motor Performance Measurement—Rotarod Test

As described previously [26], animals were placed in separate compartments on a rotating horizontal rod accelerated from 2 to 40 rpm. The time (s) was recorded when mice fell from the apparatus. The cutoff latency was 300 s. The animals were habituated to the apparatus and trained to walk during the handling procedures.

### 4.4. Pharmacological Study

#### 4.4.1. Single i.p. Administration

Cenicriviroc (ChemNorm, Wuhan, China; CVC substance with plasma half-life of 30–40 h in humans [132]) was dissolved in 1% DMSO (reconstituted in water for injection). CVC was administered once i.p. at two doses (2 and 5 mg/kg) on Day 7 after STZ injection, based on our previous studies [17]. A control group of mice was injected with vehicle (V_1_; 1% DMSO). Behavioral tests were performed 2, 4, 6, and 24 h after CVC injection.

#### 4.4.2. Repeated i.p. Administration

V_1_ (1%DMSO; solvent for CVC) or CVC (2 mg/kg) was administered i.p. twice daily, starting on Day 6 after STZ injection. Then, 0.5 h after their administration, water for injection (V_2_; solvent for morphine) or morphine (Fagron, Krakow, Poland; M; 20 mg/kg) was administered via the i.p. route. Behavioral tests were performed at several time points (on Days 8, 9, 11, 13, 15, 17, 20, and 22); the reaction was measured 0.5 h after V_2_ or M injection. Additionally, on day 21, a rotarod test was performed 0.5 h after morphine administration.

### 4.5. Biochemical Tests

Immediately after decapitation, on Day 7 post water or STZ injection, lumbar (L4–L6) spinal cords were collected after behavioral tests from non-diabetic and diabetic mice. Collected samples were subjected to biochemical analyses to assess changes in mRNA (RT-qPCR) and protein (Western blot and ELISA) levels.

#### 4.5.1. RT-qPCR

Total RNA was extracted using TRIzol reagent (Invitrogen, Carlsbad, CA, USA) according to the method reported by Chomczynski and Sacchi [133] and as described in our previous papers [26,131]. The concentration and quality of the obtained RNA were measured by a DeNovix DS-11 Spectrophotometer (DeNovix Inc., Wilmington, NC, USA). Reverse transcription was performed on 1 μg of total RNA using an Omniscript RT Kit (Qiagen Inc., Hilden, Germany), oligo (dT16) primers (Qiagen Inc., Hilden, Germany), and RNAse inhibitor (RRNasin, Promega, Mannheim, Germany). The obtained cDNA was diluted 1:10 with RNase-/DNase-free H_2_O. Approximately 50 ng of cDNA from each sample was used together with Assay-On-Demand TaqMan probes for RT-qPCR (Applied Biosystems, Foster City, CA, USA) and run on an iCycler device (Bio-Rad, Warsow, Poland). The cycle threshold values were calculated automatically by Bio-Rad CFX Manager 3.0 software using the default parameters. The following TaqMan primers were used: Mm00446968_m1 (*Hprt*, hypoxanthine-guanine phosphoribosyltransferase), Mm00441243_g1 (*Ccl2*), Mm00441259_g1 (*Ccl3*), Mm00443111_m1 (*Ccl4*), Mm0132427_m1 (*Ccl5*), Mm00443113_m1 (*Ccl7*), Mm0129783_m1 (*Ccl8*), Mm00441238_m1 (*Ccl11*), Mm0167100_m1 (*Ccl12*), Mm99999051_gH (*Ccr2*), Mm01963251_s1 (*Ccr5*), Mm0123088089 (*Oprm1*, opioid receptor mu 1), Mm01180757_m1 (*Oprd1*, opioid receptor delta 1) and Mm01230885_m1 (*Oprk1*, opioid receptor kappa 1). *Hprt1* served as a housekeeping gene for calculations. The RNA content was calculated using Formula 2−(threshold cycle).

#### 4.5.2. Western Blot

The samples were homogenized in RIPA buffer with protease inhibitor cocktail (Sigma-Aldrich, St. Louis, MO, USA) and centrifuged (14,000× *g* for 30 min). The total protein concentration was determined using the BCA Protein Assay Kit (Merck, Darmstadt, Germany). Then, the samples were diluted to 10 μg, incubated at 95 °C for 5 min with Laemmli loading buffer (Bio-Rad), and separated on 4–15% polyacrylamide Criterion™ TGX™ gels (Bio-Rad). After the semidry transfer (25 V, 30 min), the membranes (Bio-Rad) were blocked for 60 min in 5% bovine serum albumin (Sigma-Aldrich). The membranes were incubated overnight at 4 °C with primary antibodies diluted with a SignalBoost Immunoreaction Enhancer Kit (Merck), rabbit polyclonal anti-CCR5 (1:500; Novus, Abingdon, UK), rabbit polyclonal anti-CCR2 (1:1000; Novus) and mouse monoclonal anti-β-actin (1:1000; Merck) as a loading control. Next, the membranes were washed with TBST and incubated for 1 h at room temperature (RT) with HRP-conjugated secondary antibodies diluted in a SignalBoost Immunoreaction Enhancer Kit (1:5000; Vector Laboratories, Burlingame, CA, USA). Clarity™ Western ECL Substrate (Bio-Rad) was added, and target proteins were visualized using a Fujifilm LAS-4000 FluorImager system. Fujifilm Image Gauge software (Version 3.0) was used to analyze the relative levels of immunoreactivity [131].

#### 4.5.3. ELISA

The tissue was homogenized in RIPA buffer with a protease inhibitor cocktail, as described in the Section 4.5.2. ELISAs for CCL2 (ABclonal, RK00381 Woburn, MA, USA), CCL5 (ABclonal, RK00167), CCL7 (ABclonal, RK06183), and CCL8 (ABclonal, RK00425) were performed according to the manufacturer’s instructions. Samples were diluted 2–100 times and added to ELISA (Fine test, Wuhan, China) plates along with standards for 90 min at 37 °C. The plates were washed and incubated with biotin-labeled antibodies for 1 h at 37 °C. After the second wash, the samples were incubated with HRP-streptavidin conjugate for 30 min at 37 °C. The samples were washed and incubated for 15–20 min with 3,3′,5,5′-tetramethylbenzidine (TMB) at 37 °C. Then, the reaction was stopped, and the absorbance at 450 nm was measured using SPARK (Tecan, Männedorf, Switzerland) and calculated.

### 4.6. Statistical Analysis

The data from Figure 1, Figure 2, Figure 3 and Figure 4 and Table 1 are presented as the mean ± standard error of the mean (SEM) and were analyzed using Student’s *t*-tests. All biochemical assay (RT-qPCR, Western blot, and ELISA) results, except Figure 2A, which are presented as a relative expression, are presented as the fold change relative to the control (vehicle-treated non-diabetic mice). The data in Figure 5, Figure 6 and Figure 7 are shown as the mean ± SEM and the intergroup differences were analyzed using one-way analysis of variance (ANOVA) followed by Bonferroni post hoc correction for multiple comparisons. Additionally, the area under the curve (AUC) was calculated to compare the effects of the tested compounds (Figure 5 and Figure 6). Moreover, the results were evaluated using two-way ANOVA to determine the time × drug interactions (Figure 5 and Figure 6). An animal was excluded from the experiments if its behavior was abnormal or unmeasurable. All the data were visualized with GraphPad Prism 9 software (GraphPad, San Diego, CA, USA).

## 5. Conclusions

The analgesic effect of cenicriviroc, a dual CCR2/5 antagonist, relies on its ability to simultaneously block two chemokine receptors essential for nociceptive processes. Even a low (2 mg/kg) dose of CVC was able to reduce mechanical and thermal hypersensitivity in both male and female mice. Moreover, CVC has been shown to have stronger analgesic properties compared with morphine or when co-administered with morphine, which is an especially valuable datum, as the use of opioids in neuropathic pain treatments remains questionable. We believe that the results obtained in our study will contribute to the creation of an effective and safe long-term therapy based on blocking two chemokine receptors at once.

## Figures and Tables

**Table 1:** Changes in the mRNA levels of *Oprm1*, *Oprd1*, and *Oprk1* on the 7th day after vehicle or STZ treatment in male and female mice. The effects of a single STZ injection on *Oprm1*, *Oprd1,* and *Oprk1* mRNA levels were measured by RT-qPCR. The data are presented as the fold change relative to control mice (non-diabetic mice were injected with water for injection) ± SEM; total number of animals: 43 mice: male *n* = 10–12 per group; female *n* = 7–11 per group. The results were statistically evaluated using a *t*-test; ^ *p* < 0.05 and ^^ *p* < 0.01 indicate changes compared with the control. Abbreviations: STZ, streptozotocin; CTRL, control.

	MALE		FEMALE	
	CTRL	STZ	CTRL	STZ
*Oprm1*	1 ± 0.13	0.59 ± 0.07 ^	1 ± 0.09	0.85 ± 0.04
*Oprd1*	1 ± 0.19	0.35 ± 0.05 ^^	1 ± 0.20	0.74 ± 0.06
*Oprk1*	1 ± 0.10	0.80 ± 0.02	1 ± 0.33	0.70 ± 0.05

## Data Availability

The data presented in this study are available on request from the corresponding author.
